# Paracelsus on Doctors

**Published:** 1897-12

**Authors:** 


### SELECTIONS.

### Paracelsus on Doctors.

This prominent physician flourished about four hundred years ago, and his "original" ideas made him somewhat unpopular with the fraternity. He said: "he who can cure disease is a physician. Neither emperors nor popes, neither colleges nor high schools, can create physicians. They can confer special privileges and thus enable a person who is not a physician to appear as if he were one; but for all that they cannot make of him what he is not. They can give him permission to kill but they cannot enable him to cure the sick. The best of our physicians are the ones that do the least harm. But unfortunately, some poison their patients with mercury, and others purge them or bleed them to death. There are some who have learned so much that their learning has driven out all their common sense; and there are others who care a great deal more for their own profit than for the health of their patients. Medical science may be acquired by learning, but medical wisdom is the gift of God."—*Practical Druggist.*

